# Cognition, Physical Performance, and Fall-Related Mobility Outcomes in Healthy Older Adults: A Cross-Sectional Study

**DOI:** 10.3390/sports13120429

**Published:** 2025-12-03

**Authors:** Federica Marmondi, Vittoria Ferrando, Roberto Codella, Luca Filipas, Piero Ruggeri, Antonio La Torre, Emanuela Luisa Faelli, Matteo Bonato

**Affiliations:** 1 Department of Neuroscience, Rehabilitation, Ophthalmology, Genetics and Maternal Child Health, University of Genoa, 16132 Genoa, Italy; 2 Centro Polifunzionale di Scienze Motorie, University of Genoa, 16126 Genoa, Italy; vittoria.ferrando@unige.it (V.F.); ruggeri@unige.it (P.R.); emanuela.faelli@unige.it (E.L.F.); 3 Department of Experimental Medicine, Section of Human Physiology, University of Genoa, 16132 Genoa, Italy; 4 Department of Biomedical Sciences for Health, Università degli Studi di Milano, 20122 Milan, Italy; roberto.codella@unimi.it (R.C.); luca.filipas@unimi.it (L.F.); antonio.latorre@unimi.it (A.L.T.); matteo.bonato@unimi.it (M.B.); 5 Department of Endocrinology, Nutrition and Metabolic Diseases, IRCCS MultiMedica, 20138 Milan, Italy; 6 Laboratory of Movement and Sport Sciences (LaMSS), IRCCS Istituto Ortopedico Galeazzi, Via Cristina da Belgioioso 173, 20157 Milan, Italy

**Keywords:** active aging, physical fitness, cognitive performance, fall risk

## Abstract

Aging entails concurrent declines in physical and cognitive domains, contributing to reduced independence, higher fall risk, and lower quality of life. Understanding how motor performance relates to cognition is crucial for prevention in community-dwelling older adults. This cross-sectional study investigated associations between physical fitness—including strength, endurance, balance, and aerobic capacity—and cognitive outcomes assessed by Trail Making Tests (TMT-A, TMT-B), the Digit Symbol Substitution Test (DSST), and dual-task cost in the Timed Up & Go Test (TUG_DTC). Thirty-four sedentary but cognitively healthy adults aged ≥60 years were evaluated. Quadriceps strength was significantly related to TUG_DTC and executive function (TMT-B), while upper-limb endurance correlated with both attentional and executive measures. Dynamic balance, particularly in posterior directions, was linked to DSST and TMT-B, and 6-min walk test performance was associated with executive functioning and processing speed. TUG_DTC itself showed strong correlations with cognitive outcomes, underscoring its sensitivity to motor–cognitive interference. These findings highlight selective motor–cognitive relationships and suggest that simple, field-based tests may serve as early markers of cognitive vulnerability. Targeting lower-limb strength, balance, and aerobic capacity could represent cost-effective strategies to promote mobility and cognitive resilience in aging populations.

## 1. Introduction

Aging is a dynamic and multifactorial process that progressively compromises physiological integrity, functional capacity, and resilience to stressors. At the biological level, it reflects the gradual accumulation of cellular and molecular damage, which drives systemic dysregulation and increases vulnerability to a wide range of chronic conditions, including cardiovascular disease, diabetes, osteoarthritis, and neurodegenerative disorders. In addition, aging populations experience a higher prevalence of geriatric syndromes such as frailty, sarcopenia, and recurrent falls, all of which carry major consequences for autonomy and quality of life [1,2,3]. With increasing life expectancy and demographic shifts worldwide, the burden of these conditions represents a growing public health challenge.

This decline rarely affects isolated systems but often involves both physical and cognitive domains, with negative effects that tend to reinforce one another. This “dual decline” is characterized by the co-occurrence of reduced muscle strength, impaired balance, slower gait, and executive dysfunction, which together accelerate functional deterioration and increase the risk of falls, hospitalization, and long-term care admissions [4,5,6]. Falls alone remain one of the leading causes of injury-related morbidity and mortality among older adults, and they often signal underlying deficits in neuromuscular control, sensorimotor integration, and cognitive adaptability. Understanding these processes and their interactions is essential for anticipating the needs of aging societies and for designing effective preventive strategies.

To conceptually frame these associations, we refer to the Common Cause Hypothesis, which posits that age-related declines in cognitive and physical functions may stem from shared neural degeneration, particularly involving frontostriatal circuits and white matter deterioration [7,8]. This model is supported by recent neurobiological studies showing that structural brain changes and impaired cerebral perfusion can simultaneously affect motor and executive performance, especially under dual-task conditions [5,9,10].

Among the multiple domains affected by aging, physical function is often one of the first to show measurable deterioration, and it plays a critical role in determining independence. Declines in muscular strength, coordination, balance, and aerobic capacity compromise mobility, limit participation in daily activities, and reduce quality of life [11]. Importantly, inactivity acts as an accelerator of this process, leading to faster loss of muscle mass, neuromuscular efficiency, and cardiovascular function, and contributing to frailty and mortality [12]. On the other hand, structured exercise programs have been shown to mitigate these age-related impairments. For example, Ferrando et al. [13] demonstrated that a combined stretching and resistance training program improved stair descent performance, a particularly challenging and fall-prone daily task, in older women. These findings underscore the protective role of exercise and suggest that targeted interventions can preserve motor abilities and delay the onset of physical frailty.

Motor decline, however, does not occur in isolation. A growing body of evidence highlights strong interconnections between physical performance and cognitive function. Gait speed, balance, and muscle strength have been consistently associated with executive function, attention, and memory, even in otherwise healthy older adults [4,5,14,15]. Longitudinal studies further show that reduced physical fitness predicts faster cognitive decline [16,17]. Recent research reinforces this association, demonstrating how cardiorespiratory fitness, lower-limb strength, and dynamic balance are linked to attentional control, working memory, and task-switching abilities [14,15,17]. These findings suggest shared mechanisms, such as reduced cardiovascular efficiency, impaired cerebral perfusion, and white matter deterioration, which may link motor performance to cognitive outcomes. Such evidence also points to the utility of integrated assessment approaches, including dual-task paradigms, which are particularly sensitive to detecting subtle impairments across both domains and may serve as early indicators of increased risk.

Despite the growing recognition of these interrelations, important gaps remain in the literature. Most existing studies have focused on clinical populations (e.g., patients with mild cognitive impairment or Parkinson’s disease) or on intervention trials, while cross-sectional associations in sedentary but cognitively healthy older adults remain relatively unexplored [18,19,20]. Moreover, research has often concentrated on isolated parameters, such as gait speed or handgrip strength, without considering the broader network of associations between multiple domains of physical fitness and different aspects of cognition. This reductionist approach risks underestimating the complexity of interactions that shape functional performance in daily life. Furthermore, while several studies have examined individual predictors of cognitive decline, few have assessed the combined influence of multiple, ecologically valid, field-based fitness domains, including strength, endurance, dynamic balance, and dual-task mobility, on specific cognitive processes in non-clinical, sedentary aging populations [18,19,20].

To address these gaps, the present study aimed to investigate the cross-sectional associations between several domains of physical fitness (strength, endurance, dynamic balance, aerobic capacity, and dual-task performance) and cognitive outcomes (processing speed, executive function, and attention) in sedentary, cognitively healthy older adults. We hypothesized that lower performance in physical fitness tests, particularly those involving balance and dual-task mobility, would be significantly associated with poorer cognitive outcomes, especially in executive and attentional domains. By adopting a multidomain perspective, this study seeks to provide a more comprehensive understanding of the interplay between motor and cognitive health, thereby contributing to the development of targeted strategies for fall prevention and cognitive health promotion in aging populations.

## 2. Materials and Methods

### 2.1. Study Design

The present observational, cross-sectional investigation was carried out from March to July 2025 at University of Milan (Italy), adhering to the STROBE guidelines for cross-sectional studies [21]. Ethical approval was granted by the Ethical Committee of the University of Milan (protocol code: 110/24 and date of approval: 14 October 2024). All research activities respected the Declarations of Helsinki and current laws regarding human subject research. The study was registered at https://clinicaltrials.gov/ (accessed on 19 February 2025, NCT06845748). Participants were fully informed about the aims, procedures, possible risks, and benefits of the study, and provided written informed consent. The authors declare no conflicts of interest. All assessments were conducted in a single session, following a standardized and fixed order for all participants. After completing informed consent procedures, participants first underwent cognitive assessments (Trail Making Test A, Trail Making Test B, and Digit Symbol Substitution Test, in this order), followed by the physical performance assessments. The latter began with strength-related tests: Handgrip Strength Test, 30-Second Arm Curl, Quadriceps Maximal Isometric Strength, and 30-Second Chair Stand Test. Subsequently, participants completed the Timed Up and Go test (single task and then dual task), the Lower Quarter Y Balance Test, and, finally, the 6 Minute Walk Test. Adequate rest periods were provided between tests to minimize fatigue and reduce the risk of carry-over or learning effects.

### 2.2. Participants

Participants were recruited upon invitation by physicians through advertisement with posters in hospitals and online. Potential participants were screened for eligibility to assess inclusion criteria through collection of clinical history. The eligibility criteria required age 60 years or older and maintain a sedentary lifestyle, characterized by performing physical activity less than twice a week for under 20 min per session [22]. Participants were excluded if they had any medical condition requiring hospitalization within the six weeks prior to the study or if they had a history of cardiovascular diseases, musculoskeletal injuries, neurological disorders, current substance abuse, or alcohol abuse. Those who met the above inclusion criteria were invited to participate in the study. This recruitment strategy may have introduced selection bias, potentially favoring more health-conscious, motivated, or help-seeking individuals who are in regular contact with healthcare services.

### 2.3. Handgrip Strength Test

The handgrip strength (HGS) test is a measure of the maximum isometric force that a hand can squeeze [23]. The HGS was measured three times for each hand, using a digital hand dynamometer (Gripwise, Wisify Tech Solutions, Matosinhos, Portugal), a Class I medical device validated for use in older adults [24]. Subjects performed the test in a standing position, with their elbows relaxed and their wrists in a comfortable position. The subjects were instructed to squeeze the dynamometer as forcefully as possible for five seconds. A 60-s rest period was allowed between each attempt to minimize fatigue. Standardized verbal encouragement was provided during each trial to ensure maximal effort. Following three recorded trials, the mean grip strength (average of the three values) was calculated and used exclusively in the statistical analyses.

### 2.4. 30 Seconds Arm Curl

Upper body muscular strength and endurance were assessed using a modified version of the 30-s arm curl test (30sACT) [25]. This test has been validated as a reliable measure of functional upper limb strength in older adults. The test was performed using a standard chair without armrests and dumbbells weighing 2 lbs (1.8 kg) for women and 8 lbs (3.6 kg) for men, following the general setup of the original protocol. This bilateral variation was chosen to better reflect functional upper body strength during symmetrical effort, which is common in daily activities. Participants were seated upright with their feet flat on the floor, holding a dumbbell in each hand using a neutral grip. At the “go” signal provided by the trained examiner, they were instructed to perform as many full bicep curls as possible in 30 s, moving through the full range of motion (from full extension to full flexion) while keeping the upper arms close to the torso. The total number of correctly completed bilateral repetitions within 30 s was recorded for each participant, with higher scores indicating greater upper limb strength and endurance.

### 2.5. Quadriceps Maximal Isometric Strength

Quadriceps maximal isometric strength (QMIS) was assessed bilaterally using a belt-stabilized dynamometer (SAUTER FL 2K, SAUTER GmbH, Balingen, Germany), featuring a resolution of 0.1 kg and a maximum load capacity of 2000 N. Participants were seated with the knee joint positioned at a 90° angle of flexion. The use of belt-stabilized dynamometry has been demonstrated to provide valid and reliable measurements in older adults, showing strong agreement with conventional isokinetic dynamometer testing for knee extensor strength [26]. Maximal force output was recorded in Newtons (N), and three trials were conducted for each leg. Participants were instructed to maintain an upright seated posture, with their upper limbs resting on the examination table to provide trunk stability and reduce fall risk. Each effort was separated by a 30-s recovery period to minimize fatigue. Standardized verbal encouragement was given throughout the test to promote maximal voluntary contraction. The mean value of the three trials for each leg was calculated and subsequently used for statistical analyses.

### 2.6. 30 Seconds Chair Stand Test

The 30 s chair stand test (30sCST) was performed to evaluate lower limbs strength [25,27]. The test has been shown to provide valid and reliable data on leg strength in older adults. The CST was administered using a chair with a seat height of 17 inches (43.2 cm) and the subject had to start the test in a sitting position with crossed arms at the wrists and head on the chest. At the “go” signal by the expert investigator, the participants had to rise in a full standing position and then return to the initial sitting position. The subject had to complete as many full stands as possible in 30 s and higher scores indicate higher lower limb strength values.

### 2.7. Timed up and Go Test

Functional mobility and dual-task performance were assessed using both the Timed Up and Go (TUG_ST) test and its dual-task variation (TUG_DT), following the procedures described in the Mini-Balance Evaluation Systems Test [28]. The standard TUG, originally developed by Podsiadlo and Richardson [29], was administered first to assess basic mobility. Participants were asked to stand up from a standard armchair, walk 3 m at a comfortable pace, turn around a cone, return, and sit down. The total completion time (in seconds) was recorded. This test is not only widely used in clinical settings but has also demonstrated predictive validity for fall risk in community-dwelling older adults [18]. In the dual-task condition, participants performed the same task while simultaneously subtracting by threes from a random number. No verbal encouragement or feedback was given. If the subtraction sequence was incorrect, the trial was repeated using a different starting number. However, the number of correct responses and cognitive task accuracy were not recorded, and only motor performance was analyzed. For statistical analysis, only the completion time (in seconds) was considered for both the single-task and dual-task conditions. Additionally, the dual-task cost (DTC) was calculated as the difference in time between the TUG_DT and the standard TUG, providing a quantitative measure of the cognitive interference on mobility. Higher TUG_DTC values indicate greater cognitive-motor interference. This measure reflects the extent to which cognitive load affects gait and mobility and has been linked to fall risk and executive function impairment [5,30].

### 2.8. Lower Quarter Y Balance Test

Dynamic balance of the lower limbs was evaluated using the Lower Quarter Y Balance Test (YBT-LQ), a validated and widely adopted assessment tool for identifying deficits in postural control and asymmetries in reach performance across the lower extremities [31]. This protocol represents an enhanced version of the original Star Excursion Balance Test, designed to produce standardized and repeatable measurements in three directions: anterior, posteromedial, and posterolateral. Testing followed a structured and previously validated procedure [31,32]. The YBT-LQ was conducted using a dedicated kit that included a stance platform with three polyvinyl chloride reach pipes positioned in the anterior (ANT), posteromedial (PM), and posterolateral (PL) directions. The posterior arms were placed at 135° relative to the anterior, with a 45° separation between them. Each pipe featured 5 mm measurement increments for accuracy.

During testing, participants placed the distal end of their great toe at the intersection point of the Y-configuration and extended the opposite limb to reach in each of the three target directions, pushing a standardized indicator to mark the reach distance. None of the subjects had prior experience with the YBT-LQ, which minimized any potential learning effects. Following a video-based instructional session, participants received standardized instructions and were given six practice trials per direction, in line with established testing guidelines [31]. The first three trials in each direction were used for familiarization, while the final three attempts were recorded and included in the analysis. To ensure consistency, trials were deemed invalid if the reach foot contacted the ground, the balance indicator, or the support structure. In such cases, participants repeated the attempt up to a total of six tries. If unable to meet criteria, no score was assigned for that direction.

Reach distances were calculated from the distal end of the stance foot’s toes to the furthest point reached by the opposite limb. For each leg and direction, the average of the final three successful trials was used in subsequent analyses. Additionally, a composite score (YBT-CS) was derived following the formula proposed by Plisky et al. [29]: [(ANT + PM + PL)/(3 × LL)] × 100. Maximum values for each direction and the combined composite score per limb were also examined. To account for inter-individual differences in limb length, all YBT-LQ results were normalized relative to the participant’s average anatomical limb length (mean of right and left limbs).

### 2.9. Six Minutes Walking Test

Six Minutes Walking Test (6MWT) is a validated submaximal test aimed to assess cardiorespiratory fitness in pathological population [33]. 6MWT was assessed in a 30-m indoor corridor with participants that were instructed to walk as fast as possible for six min. Total distance at the end of the test was recorded.

### 2.10. Cognitive Assessments

#### 2.10.1. Trail Making Tests

The Trail Making Test (TMT) consists of a freely available, timed, neuropsychological test, formed by 2 parts, that involves visual scanning and working memories [34]. It is commonly used to assess processing speed, attention, and executive functions in aging populations. Trail Making Test A (TMT-A) is formed by randomly positioned circled numbers that subject had to connect as quickly as possible in numeric order. TMT-A primarily evaluates visual scanning, psychomotor speed, and sustained attention. Trail Making Test B (TMT-B) consists of randomly positioned circled numbers and letters. The subject had to connect as quickly as possible the circles in numeric and alphanumeric order, alternating between numbers and letters. TMT-B requires additional cognitive demands, especially task-switching, divided attention, and cognitive flexibility, and is considered more sensitive to early executive function decline. The TMT was scored by how long it took to complete the test. The time included correction of errors prompted by the examiner. If the subject did not complete the test in 5 min, the test was discontinued.

#### 2.10.2. Digit Symbol Substitution Test

The Digit Symbol Substitution Test (DSST) consists in a freely available, timed, neuropsychological test [35]. In DSST, the subject had to fill in a series of symbols correctly coded within 90 s. The score was the number of correct number-symbol matches achieved in 90 s. The DSST evaluated multiple cognitive domains, including processing speed, sustained attention, visual-motor coordination, and working memory. It is considered a sensitive marker of general cognitive efficiency and is frequently used in studies of aging, dementia risk, and functional decline.

### 2.11. Statistical Analysis

This cross-sectional analysis was conducted using baseline data from a randomized controlled trial involving 34 participants. The sample size was determined a priori based on the primary objective of the trial, which aimed to detect pre–post intervention effects with a medium effect size (f = 0.25), α = 0.05, and power (1 − β) = 0.80. Although this power analysis was not designed for cross-sectional correlational analyses, the same sample was considered appropriate for exploratory assessment of baseline associations between physical and cognitive functions. Specifically, the Timed Up & Go test with dual task was selected as the primary mobility variable, as it has been shown to increase the sensitivity of the Timed Up & Go test in detecting subtle impairments in cognitive–motor performance [30]. Previous literature supports moderate to strong correlations between dual-task Timed Up & Go performance and executive function in older adults [19,36].

Quantitative variables were expressed as mean ± standard deviation (SD). The distribution of each variable was assessed through graphical inspection and the Shapiro–Wilk test to verify the assumption of normality. As all variables were normally distributed, Pearson’s correlation coefficient (r) was used to explore the relationships between functional and cognitive performance measures. The strength of correlation was interpreted as follows: 0.00–0.30 = negligible, 0.30–0.50 = low, 0.50–0.70 = moderate, 0.70–0.90 = high, 0.90–1.00 = very high [37]. In this exploratory analysis, correlations with r > 0.30 and *p* < 0.05 were interpreted as statistically significant, based on conventional thresholds for low but potentially relevant associations in clinical research [37].

For each correlation, the corresponding effect size was expressed as the coefficient of determination (R^2^), and post hoc statistical power (1 − β) was calculated using G*Power 3.1 [38], based on the observed r values, α = 0.05, and total sample size (N = 34). In addition, 95% confidence intervals (95% CI for R) for each correlation coefficient were reported to improve interpretability. Given the large number of correlations performed, we acknowledged the increased risk of Type I error ±due to multiple testing. Therefore, false discovery rate (FDR)-adjusted *p*-values were calculated using the Benjamini–Hochberg procedure (Q = 0.05), and are reported as q-values in Supplementary Table S1.

While FDR correction was applied to control for false discoveries, the primary interpretation of results was based on unadjusted *p*-values, consistent with the exploratory aims of the study. Effect sizes and confidence intervals were also considered to support the relevance of findings.

Statistical analyses were performed using GraphPad Prism, version 10.6.0 (GraphPad Software, San Diego, CA, USA).

## 3. Results

The study included 34 participants who met the eligibility criteria. Baseline characteristics of the sample are presented in Table 1. The sample consisted of 22 men (65%) and 12 women (35%).

The associations between functional capacity and cognitive measures were examined using Pearson’s correlation (R) and are detailed in Table 2, which also reports corresponding *p*-values (*p*), linear regression equations (Y), coefficients of determination (R^2^), and post hoc statistical power estimates (1 − β).

**Figure 1:**
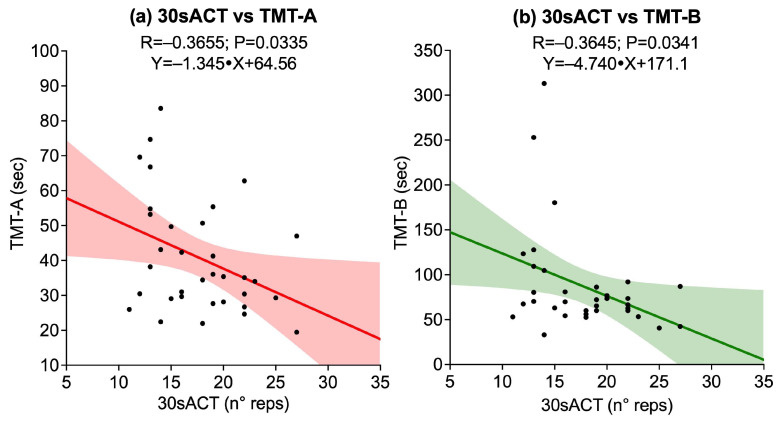
Scatter plots showing significant associations between upper-limb strength tests and cognitive outcomes: (**a**) 30-second Arm Curl Test (30sACT) vs. Trail Making Test A (TMT-A); (**b**) 30sACT vs. Trail Making Test B (TMT-B). Solid lines represent linear regression fits, and shaded areas indicate 95% confidence intervals.

Correlations between upper-limb strength tests, assessed through right and left HGS and 30sACT, and cognitive performance on TMT-A, TMT-B, and DSST revealed no significant associations between right HGS and any cognitive outcome [TMT-A (*p* = 0.8500), TMT-B (*p* = 0.4262), DSST (*p* = 0.6669)]. Similarly, left HGS was not significantly correlated with any cognitive outcome [TMT-A (*p* = 0.2638), TMT-B (*p* = 0.2702), DSST (*p* = 0.3519)]. Conversely, 30sACT showed significant negative correlations with both TMT-A (R = −0.3655, *p* = 0.0335) and TMT-B (R = −0.3645, *p* = 0.0341), while its correlation with DSST did not reach statistical significance (*p* = 0.0112). Significant associations are illustrated in Figure 1.

**Figure 2:**
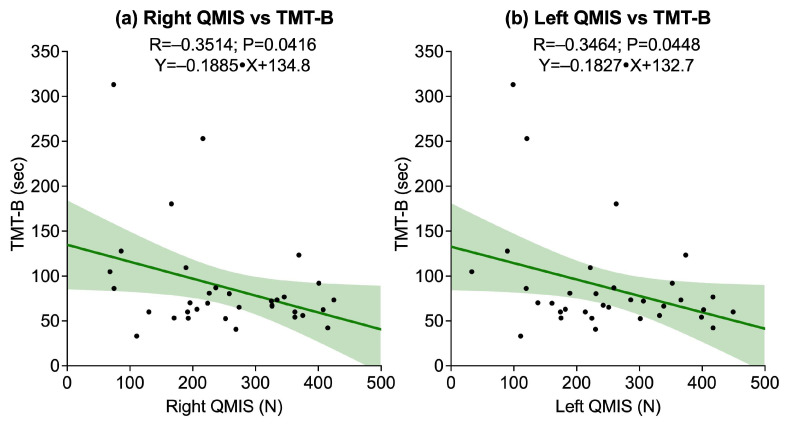
Scatter plots showing associations between quadriceps muscle isometric strength (QMIS) and cognitive outcome assessed by the Trail Making Test B (TMT-B): (**a**) Right QMIS vs. TMT-B; (**b**) Left QMIS vs. TMT-B. Solid lines represent linear regression fits, and shaded areas indicate 95% confidence intervals.

The correlations between lower-limb strength tests, assessed through right and left QMIS and 30sCST, and cognitive performance on TMT-A, TMT-B, and DSST revealed several significant associations. A statistically significant negative correlation was found between right QMIS and TMT-B (R = −0.3514, *p* = 0.0416), while the correlations with TMT-A (*p* = 0.0704) and DSST (*p* = 0.1558) were not significant. Similarly, left QMIS was significantly associated with TMT-B (R = −0.3464, *p* = 0.0448), whereas no significant associations emerged with TMT-A (*p* = 0.0632) or DSST (*p* = 0.2132). Conversely, 30sCST did not show significant correlations with any of the cognitive outcomes: TMT-A (*p* = 0.0864), TMT-B (*p* = 0.1059), or DSST (*p* = 0.3620). Figure 2 illustrates the significant associations.

**Figure 3:**
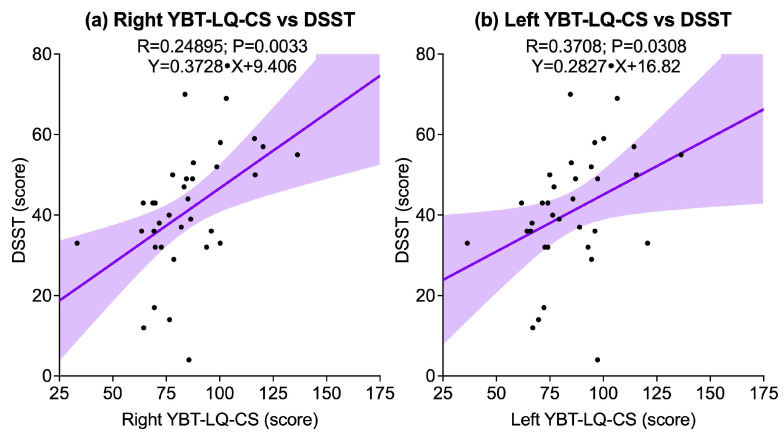
Scatter plots illustrating significant associations between the Y-Balance Test for the Lower Quarter composite score (YBT-LQ-CS) and cognitive outcomes assessed by the Digit Symbol Substitution Test (DSST): (**a**) Right YBT-LQ-CS vs. DSST; (**b**) Left YBT-LQ-CS vs. DSST. Solid lines represent linear regression fits, and shaded areas indicate 95% confidence intervals.

Dynamic balance, as assessed through YBT-LQ-CS, showed significant associations with cognitive performance. A positive correlation was observed between right YBT-LQ-CS and DSST (R = 0.24895, *p* = 0.0033), while a weaker but still significant correlation was also found between left YBT-LQ-CS and DSST (R = 0.3708, *p* = 0.0308). No significant correlations emerged between either side and TMT-A (right: *p* = 0.2745; left: *p* = 0.1834) or TMT-B (right: *p* = 0.1982; left: *p* = 0.1600). Figure 3 illustrates the significant associations.

**Figure 4:**
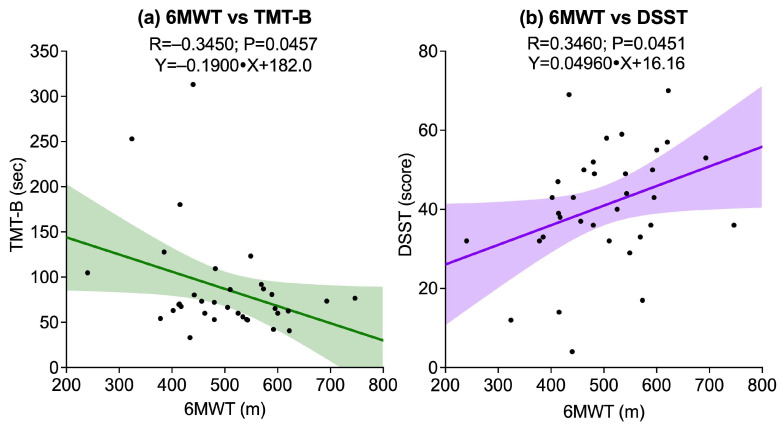
Scatter plots showing associations between the Six-Minute Walk Test (6MWT) and cognitive outcomes: (**a**) 6MWT vs. Trail Making Test B (TMT-B); (**b**) 6MWT vs. Digit Symbol Substitution Test (DSST). Solid lines represent linear regression fits, and shaded areas indicate 95% confidence intervals.

Regarding functional exercise capacity, measured 6MWT, a significant negative correlation was found with TMT-B (R = −0.3450, *p* = 0.0457), and a significant positive correlation was observed with DSST (R = 0.3460, *p* = 0.0451). The correlation with TMT-A did not reach statistical significance (*p* = 0.0638). Figure 4 illustrates these significant associations.

**Figure 5:**
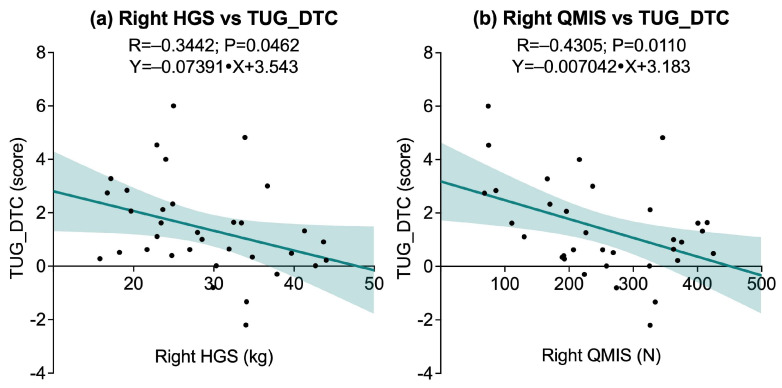
Scatter plots showing significant associations between dual-task cost in the Timed Up and Go test (TUG_DTC) and physical performance variables: (**a**) Right Handgrip Strength (HGS) vs. TUG_DTC; (**b**) Right Quadriceps Muscle Isometric Strength (QMIS) vs. TUG_DTC. Solid lines represent linear regression fits, and shaded areas indicate 95% confidence intervals.

The associations between physical performance variables and cognitive–motor interference, as measured by the dual-task cost in the Timed Up & Go test (TUG_DTC), revealed a selective pattern. A significant negative correlation was found between right HGS R = −0.3442, *p* = 0.0462), while the correlation with left HGS did not reach statistical significance (*p* = 0.0576). Similarly, right QMIS was significantly associated (R = −0.4305, *p* = 0.0110), whereas no significant association was observed for left QMIS (*p* = 0.1182). No significant correlations were found between TUG_DTC and muscular endurance measures, including 30sACT (*p* = 0.8171) and 30sCST (*p* = 0.5616). Additionally, no significant associations emerged between TUG_DTC and dynamic balance performance, as assessed by right and left YBT-LQ-CS (*p* = 0.4875 and *p* = 0.6995, respectively). Functional exercise capacity, measured by the 6MWT, also showed no significant correlation with TUG_DTC (*p* = 0.5104). Figure 5 illustrates the significant associations.

**Figure 6:**
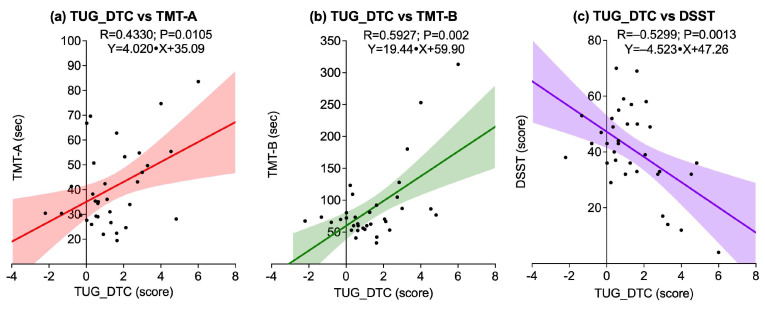
Scatter plots showing associations between dual-task cost in the Timed Up and Go test (TUG_DTC) and cognitive outcomes: (**a**) TUG_DTC vs. Trail Making Test A (TMT-A); (**b**) TUG_DTC vs. Trail Making Test B (TMT-B); (**c**) TUG_DTC vs. Digit Symbol Substitution Test (DSST). Solid lines represent linear regression fits, and shaded areas indicate 95% confidence intervals.

Figure 6 illustrates the associations between TUG_DTC and all cognitive tests. A statistically significant positive correlation was observed between TUG_DTC and TMT-A (R = 0.4330; *p* = 0.0105), as well as between TUG_DTC and TMT-B (R = 0.5927; *p* = 0.0020), indicating that greater dual-task cost was associated with longer completion times on both TMTs. Conversely, a significant negative correlation was found between TUG_DTC and DSST (R = −0.5299; *p* = 0.0013), suggesting that higher TUG_DTC scores were associated with lower DSST performance.

## 4. Discussion

The present study examined how different physical performance domains [maximal isometric strength (HGS and QMIS), muscular endurance (30sACT and 30sCST), dynamic balance (YBT-LQ in multiple directions), and function exercise capacity (6MWT)] relate to cognitive outcomes assessed by TMT-A, TMT-B, DSST, and dual task cost in Timed Up & Go Test (TUG_DTC). Overall, the results support the interdependence of physical and cognitive functioning in aging, highlighting selective relationships between motor abilities and specific cognitive domains. This contributes to the growing body of literature suggesting that physical performance is not only a determinant of functional independence but also a potential marker of cognitive resilience in older adults.

In the strength domain, QMIS was significantly correlated with dual-task cost and executive functioning (TMT-B), but not with processing speed (TMT-A and DSST). These findings extend previous research showing that lower-limb strength predicts dual-task gait performance and fall risk [5,39], suggesting that muscular strength may buffer cognitive–motor interference and sustain higher-order processes such as task-switching and attentional flexibility. The selective association with executive function, but not with basic attention or processing speed, points to a domain-specific link between lower-limb force production and cognitive control mechanisms. Interestingly, handgrip strength showed weaker and lateralized associations, with significant results only for the dominant hand, which correlated with TUG_DTC. This asymmetry may reflect limb dominance effects, consistent with previous reports that dominant-limb strength could be more closely related to certain neurocognitive processes [9,40], although this interpretation remains tentative. The lack of association with processing speed measures further suggests that strength is not uniformly related to cognition but may preferentially support executive and integrative domains. These observations highlight the clinical relevance of incorporating lower-limb strength testing into geriatric screening, as it may potentially indicate combined changes in mobility and executive control decline, although this interpretation should be considered preliminary and requires further confirmation. These findings align with previous evidence supporting the predictive value of lower-limb strength for mobility and executive function in older adults [39,41], yet they also highlight a potentially greater sensitivity of isolated quadriceps strength over global functional strength measures such as the 30sCST. This may be due to the biomechanical specificity of QMIS, which may better capture aspects of neuromuscular capacity and motor unit recruitment, whereas the 30sCST is influenced by multiple factors including balance, speed, and motivation, which could dilute its association with cognitive processes. Furthermore, the robust relationship observed between TUG_DTC and cognitive tests, particularly TMT-B and DSST, reinforces the literature indicating that dual-task paradigms are more sensitive to early cognitive–motor decline than single-task assessments [5,42]. These patterns support the integration of both lower-limb strength and dual-task mobility in cognitive screening protocols for aging populations.

Regarding muscular endurance, the results revealed a differential pattern. The 30 s Arm Curl Test was significantly correlated with both TMT-A and TMT-B, whereas the 30 s Chair Stand Test showed no significant associations. This suggests that repetitive upper-limb movements requiring pacing, coordination, and attentional engagement may more directly tap cognitive resources than sit-to-stand movements, which rely more heavily on automatic motor patterns and postural stability. Prior studies have reported similar findings, emphasizing that upper-limb endurance tasks are sensitive to sustained attention and executive function [8,10]. The lack of associations for the 30sCST could be explained by ceiling effects, as many participants in healthy samples achieve high repetition counts with limited variability [43], thereby reducing sensitivity to cognitive differences. Alternatively, sit-to-stand performance may be influenced more by muscle power, joint mobility, or balance strategies, rather than cognitive control [5]. Taken together, these results underscore the value of considering task-specific cognitive demands when selecting endurance measures for cognitive screening purposes.

Although both QMIS and 30sCST assess aspects of lower-limb performance, they are not redundant. QMIS reflects isolated muscle strength under controlled, isometric conditions, whereas the 30sCST captures dynamic endurance and functional capacity in a multi-joint task. The inclusion of both measures allows for a more nuanced characterization of physical function [44,45]. However, in this sample, the stronger associations observed for QMIS may suggest that maximal strength is a more sensitive correlate of executive function, although this interpretation should be considered exploratory [46].

Dynamic balance, as assessed through the multidirectional Y-Balance Test, showed robust associations with both DSST and TMT-B, reinforcing the evidence that postural control is cognitively demanding and relies on executive and attentional resources [47,48]. Notably, posterior reach directions (PM and PL) appeared particularly sensitive, suggesting that challenging balance adjustments in these directions require greater neuromuscular coordination and attentional allocation [49,50]. In contrast, TUG_DTC was not significantly related to YBT components, indicating that dual-task mobility and anticipatory balance control may rely on distinct cognitive–motor mechanisms. These findings align with previous evidence of dissociation between balance and gait-related dual-task costs, suggesting that different neural circuits underlie static or anticipatory versus dynamic locomotor control. Such domain-specific associations support the inclusion of multidirectional balance testing in comprehensive geriatric assessments, as it may reveal subtle cognitive–motor links not captured by global mobility measures.

Functional exercise capacity, measured by the 6MWT, was significantly associated with executive function (TMT-B) and processing speed (DSST), but not with TMT-A or TUG_DTC. This is consistent with prior evidence showing that aerobic fitness supports higher-order cognitive processes, particularly cognitive flexibility and processing efficiency [51,52,53,54]. The absence of associations with TUG_DTC suggests that aerobic capacity alone may not buffer cognitive–motor interference during dual-task walking, which may depend more on attentional control and neuromotor integration [42,55]. These findings suggest that while aerobic endurance contributes to overall cognitive health, targeted dual-task training may be required to reduce interference during complex mobility tasks. The relatively weaker associations observed for the 6MWT with cognitive outcomes may reflect a ceiling effect in this healthy, sedentary sample, where most participants achieved satisfactory performance levels with limited interindividual variability [27,56]. Alternatively, aerobic endurance may contribute more to cognitive maintenance over time rather than being directly associated in cross-sectional designs [53,57]. This suggests the need for longitudinal analyses to clarify whether aerobic capacity predicts cognitive trajectories rather than static performance.

Finally, TUG_DTC itself was associated with poorer performance on TMT-A and TMT-B, but positively correlated with DSST, reinforcing its sensitivity as a marker of executive efficiency and attentional allocation under cognitive load [5,42]. These results highlight the clinical value of dual-task paradigms as sensitive indicators of cognitive–motor interference, even in sedentary but cognitively intact older adults, and suggest their potential use in early screening for subtle decline.

Despite the relevance of these findings, several limitations must be acknowledged. The cross-sectional design prevents causal inference, and the relatively small and homogeneous sample (N = 34), though determined a priori within a randomized controlled trial framework, limits statistical power and generalizability. Additionally, the limited sample size raises the possibility of Type II errors, meaning that some true associations may have gone undetected. Conversely, the number of statistical tests conducted increases the risk of overfitting, whereby certain findings may reflect sample-specific noise rather than robust effects. These concerns further support a cautious and exploratory interpretation of the results. The observed associations should thus be interpreted as exploratory. Furthermore, important confounding variables, such as comorbidities, medication use, and psychosocial factors, were not assessed. In particular, the absence of standardized screening for depressive symptoms, known to affect executive functioning and attention, may have influenced the results. Sociodemographic and health-related factors (e.g., education, smoking, comorbidity burden) were also not systematically collected, preventing their inclusion as covariates. Future studies should account for these variables to improve internal validity. Additionally, possible ceiling effects in endurance tests may have reduced sensitivity to cognitive associations. Moreover, although a dual-task paradigm was implemented, only motor performance (TUG time) was recorded, while cognitive task accuracy during the subtraction task was not measured. This limits the interpretation of cognitive–motor interference, as the cognitive cost could not be quantified. Not all cognitive domains were assessed, which may limit interpretability. A more comprehensive neuropsychological batter, including measures of language, visuospatial abilities, and episodic memory, would help capture a broader picture of cognitive function. While these findings are promising, replication in larger, more diverse samples and longitudinal or interventional studies is needed to confirm and extend the current results. Future research should also examine whether improvements in specific physical domains (e.g., strength, aerobic endurance) lead to cognitive benefits, particularly in executive and dual-task performance. Integrating biomarkers and neuroimaging techniques may further clarify the mechanisms underlying motor–cognitive relationships and support the development of targeted interventions for healthy aging.

## 5. Conclusions

In conclusion, the present study contributes to the growing body of evidence linking physical performance with cognitive function in aging, particularly under dual-task conditions that reflect real-life challenges. Among the physical fitness parameters examined, lower-limb strength emerged as the most robust correlate of dual-task cost, while aerobic capacity was associated with executive function and processing speed. These findings underscore the importance of evaluating and targeting specific physical domains, especially muscular strength and cardiorespiratory fitness, in clinical assessments and intervention programs aimed at preserving cognitive health, mobility, and independence in older adults.

## Figures and Tables

**Table 1:** Baseline characteristics of the 34 subjects who participated in the study.

	Mean ± SD	Median (Q1–Q3)	Min–Max	95% CI
Age (years)	70 ± 7	69 (65–76)	60–84	67.79–72.62
Height (m)	1.70 ± 0.09	1.70 (1.62–1.75)	1.52–1.90	1.67–1.73
BMI (kg/m^2^)	23.6 ± 3.2	22.7 (21.2–26.0)	17.8–29.4	22.4–24.6
Right HGS—mean (kg)	28.9 ± 8.2	28.3 (22.9–34.3)	15.8–44.0	23.1–31.8
Left HGS—mean (kg)	25.2 ± 7.6	24.3 (18.7–32.8)	13.8–41.0	22.5–27.8
30sACT (n° reps)	18 ± 4	18 (14–22)	11–27	16.2–19.3
Right QMIS—mean (N)	252.6 ± 107.5	244.5 (184.6–349.9)	68.3–424.7	215.1–290.1
Left QMIS—mean (N)	249.1 ± 109.4	236.5 (170.7–342.1)	33.0–449.3	210.9–287.2
30sCST (n° reps)	16 ± 5	15 (12–18)	3–29	13.7–17.3
6MWT (m)	499.1 ± 104.7	493.5 (416.5–577.0)	240.0–746.5	462.6–535.7
Right YBT-LQ-CS—mean (score)	84.5 ± 19.7	83.6 (69.8–96.7)	33.3–136.4	77.6–91.4
Left YBT-LQ-CS—mean (score)	85.2 ± 19.7	84.8 (72.0–96.4)	36.4–136.4	78.3–92.1
Right YBT-LQ-ANT (cm)	73.5 ± 15.2	68.8 (63.3–86.8)	30.0–101.7	68.2–78.8
Right YBT-LQ-PM (cm)	76.7 ± 17.3	79.2 (64.2–86.0)	36.7–114.3	70.7–82.7
Right YBT-LQ-PL (cm)	79.1 ± 19.4	80.4 (65.6–93.3)	36.7–116.7	72.4–85.9
Left YBT-LQ-ANT (cm)	74.3 ± 13.7	72.2 (66.7–86.1)	31.7–99.2	69.5–79.0
Left YBT-LQ-PM (cm)	77.2 ± 16.4	80.0 (62.5–88.3)	38.3–105.0	71.5–82.9
Left YBT-LQ-PL (cm)	80.5 ± 18.4	84.6 (64.3–92.5)	38.3–116.7	74.1–86.9
TUG_ST (s)	7.7 ± 1.4	7.4 (7.0–8.3)	5.4–11.3	7.2–8.2
TUG_DT (s)	9.1 ± 2.5	8.6 (7.2–10.1)	5.7–15.0	8.3–10.0
TUG_DTC (score)	1.4 ± 1.8	1.1 (0.3–2.4)	−2.2–6.0	0.8–2.0
TMT-A (s)	40.7 ± 16.3	35.2 (28.8–51.3)	19.5–83.6	35.0–46.4
TMT-B (s)	87.2 ± 57.7	70.1 (59.0–88.2)	33.0–313.1	67.1–107.3
DSST (score)	41 ± 15	42 (33–51)	4–70	35.7–46.2

Values are expressed as mean ± standard deviations (SD), median (Q1–Q3), minimum and maximum (Min–Max), and 95% confidence intervals (CI). Legend: BMI: body mass index; HGS: HandGrip Strength test; 30sACT: 30-s Arm Curl Test; QMIS: Quadriceps Maximal Isometric Strength; 30sCST: 30-s Chair Stand Test; 6MWT: 6 Minutes Walking Test; YBT-LQ-CS: Y-Balance Test-Lower Quarter-Composite Score; YBT-LQ-ANT: Y-Balance Test-Lower Quarter-Anterior; YBT-LQ-PM: Y-Balance Test-Lower Quarter-PosteroMedial; YBT-LQ-PL: Y-Balance Test-Lower Quarter-PosteroLateral; TUG_ST: Timed Up & Go test Single Task; TUG_DT: Timed Up & Go test Dual Task; TUG_DTC: Timed Up & Go Test Dual Task Component; TMT-A: Trail Making Test A; TMT-B: Trail Making Test B; DSST: Digit Symbol Substitution Test.

**Table 2:** Correlation coefficients (R), significance levels (*p*), and linear regression models (Y) between functional performance tests and cognitive/mobility measures (N = 34).

		TMT-A (s)	TMT-B (s)	DSST (Score)	TUG_ST (s)	TUG_DT (s)	TUG_DTC (Score)
	
Right HGS (kg)	R	−0.03368	−0.1411	0.07658	−0.3722	−0.4563	−0.3442
	95% CI for R	−0.3677 to 0.3080	−0.4574 to 0.2070	−0.2685 to 0.4043	−0.6310 to −0.03895	−0.6883 to −0.1397	−0.6112 to −0.006845
	*p*	0.8500	0.4262	0.6669	0.0302	0.0067	0.0462
	Y	−0.06713 × X + 42.68	−0.9932 × X + 115.9	0.1404 × X + 36.85	−0.06379 × X + 9.561	−0.1373 × X + 13.10	−0.07391 × X + 3.543
	R^2^	0.0011	0.0199	0.0059	0.1385	0.2082	0.1185
	1 − β	0.0540	0.1249	0.0713	0.0533	0.7955	0.5259
							
Left HGS (kg)	R	−0.1971	−0.1946	0.1647	−0.3277	−0.4201	−0.3288
	95% CI for R	−0.5019 to 0.1511	−0.4999 to 0.1537	−0.1837 to 0.4764	−0.5995 to 0.01174	−0.6639 to −0.09550	−0.6002 to 0.01055
	*p*	0.2638	0.2702	0.3519	0.0585	0.0134	0.0576
	Y	−0.4233 × X + 51.40	−1.476 × X + 124.3	0.3252 × X + 32.72	−0.06012 × X + 9.240	−0.1362 × X + 12.56	−0.07605 × X + 3.319
	R^2^	0.0389	0.0379	0.0271	0.1074	0.1765	0.1081
	1 − β	0.2016	0.1975	0.1537	0.4837	0.7160	0.4865
							
30sACT (n° reps)	R	−0.3655	−0.3645	0.2782	−0.3504	−0.1689	0.04119
	95% CI for R	−0.5995 to 0.01174	−0.6256 to −0.03003	−0.06624 to 0.5633	−0.6156 to −0.01390	−0.4797 to 0.1795	−0.3012 to 0.3741
	*p*	0.0335	0.0341	0.1112	0.0422	0.3395	0.8171
	Y	−1.345 × X + 171.1	−4.740 × X + 171.1	0.9415 × X + 24.24	−0.1102 × X + 9.678	−0.09389 × X + 10.79	0.01633 × X + 1.115
	R^2^	0.1336	0.1329	0.0774	0.1228	0.0285	0.0017
	1 − β	0.5807	0.5782	0.3628	0.5419	0.1593	0.0560
							
Right QMIS (N)	R	−0.3141	−0.3514	0.2489	−0.2059	−0.4237	−0.4305
	95% CI for R	−0.5897 to 0.02689	−0.6163 to −0.01499	−0.09747 to 0.5415	−0.5086 to 0.1422	−0.6664 to −0.09990	−0.6710 to −0.1081
	*p*	0.0704	0.0416	0.1558	0.2427	0.0125	0.0110
	Y	−0.04770 × X + 52.79	−0.1885 × X + 134.8	0.03475 × X + 32.14	−0.002671 × X + 8.401	−0.009713 × X + 3.183	−0.007042 × X + 3.183
	R^2^	0.0987	0.1235	0.0620	0.0424	0.1796	0.1854
	1 − β	0.4494	0.5444	0.2982	0.5419	0.7244	0.7400
							
Left QMIS (N)	R	−0.3222	−0.3464	0.2191	−0.02172	−0.3177	−0.2730
	95% CI for R	−0.5954 to 0.01797	−0.6128 to −0.009302	−0.1286 to 0.5188	−0.5173 to 0.1306	−0.5923 to 0.02288	−0.5595 to 0.07175
	*p*	0.0632	0.0448	0.2132	0.2173	0.0671	0.1182
	Y	−0.04809 × X + 52.72	−0.1827 × X + 132.7	0.03007 × X + 33.42	−0.002770 × X + 8.416	0.007161 × X + 10.91	−0.004391 × X + 2.498
	R^2^	0.1038	0.1200	0.0480	0.0471	0.1010	0.0746
	1 − β	0.4698	0.5316	0.2397	0.2362	0.4584	0.3509
							
30sCST (n° reps)	R	−0.2985	−0.2821	0.1613	−0.3666	−0.2811	−0.1031
	95% CI for R	−0.5783 to 0.04416	−0.5663 to 0.06192	−0.1870 to 0.4737	−0.6270 to −0.03246	−0.5655 to 0.06306	−0.4264 to 0.2435
	*p*	0.0864	0.1059	0.3620	0.0330	0.1073	0.5616
	Y	−0.9328 × X + 55.20	−3.115 × X + 135.5	0.4636 × X + 33.73	−0.09790 × X + 9.244	−0.1326 × X + 11.19	−0.03473 × X + 1.942
	R^2^	0.0891	0.0796	0.0260	0.1344	0.0790	0.0106
	1 − β	0.4109	0.3419	0.1492	0.5835	0.3696	0.0890
							
6MWT (m)	R	−0.3214	−0.3450	0.3460	−0.5138	−0.3742	−0.1169
	95% CI for R	−0.5949 to 0.01876	−0.6118 to −0.007742	0.008818 to 0.6125	−0.7258 to −0.2125	−0.6323 to −0.04124	−0.4377 to 0.2304
	*p*	0.0638	0.0457	0.0451	0.0019	0.0292	0.5104
	Y	−0.05012 × X + 65.75	−0.1900 × X + 182.0	0.04960 × X + 16.16	−0.006845 × X + 11.14	−0.008807 × X + 13.53	−0.001963 × X + 2.384
	R^2^	0.1033	0.1190	0.1197	0.2640	0.1400	0.0137
	1 − β	0.4677	0.5280	0.5303	0.8945	0.6030	0.1006
							
Right YBT-LQ-CS (score)	R	−0.3333	−0.2738	0.4895	−0.2371	−0.2221	−0.1232
	95% CI for R	−0.6034 to 0.005510	−0.5601 to 0.07088	0.1814 to 0.7101	−0.5326 to 0.1099	−0.5211 to 0.1255	−0.4429 to 0.2243
	*p*	0.0541	0.1171	0.0033	0.1770	0.2068	0.4875
	Y	−0.2760 × X + 64.07	−0.8011 × X + 154.9	0.3728 × X + 9.406	−0.01678 × X + 9.144	−0.02777 × X + 11.48	−0.01099 × X + 2.333
	R^2^	0.1111	0.0750	0.2396	0.0562	0.0493	0.0152
	1 − β	0.4980	0.3527	0.0500	0.2742	0.2452	0.1065
							
Left YBT-LQ-CS (score)	R	−0.1561	−0.1679	0.3708	−0.2573	−0.1947	−0.06868
	95% CI for R	−0.4695 to 0.1922	−0.4789 to 0.1805	0.03735 to 0.6300	−0.5478 to 0.08852	−0.4999 to 0.1536	−0.3976 to 0.2759
	*p*	0.3760	0.3426	0.0308	0.1417	0.2699	0.6995
	Y	−0.1294 × X + 5177	−0.4916 × X + 129.1	0.2827 × X + 16.82	−0.01823 × X + 9.279	−0.02436 × X + 11.21	−0.006133 × X + 1.927
	R^2^	0.0244	0.0282	0.1375	0.0662	0.0379	0.0662
	1 − β	0.1426	0.1580	0.5943	0.3161	0.1977	0.0670
							
Right YBT-LQ-ANT (cm)	R	−0.2044	−0.1617	0.3336	−0.2769	−0.2324	−0.1061
	95% CI for R	−0.5075 to 0.1437	−0.4740 to 0.1866	−0.005111 to 0.6037	−0.5624 to 0.06762	−0.5290 to 0.1147	−0.4289 to 0.2407
	*p*	0.2462	0.3608	0.0538	0.1129	0.1859	0.5502
	Y	−0.2194 × X + 56.86	−0.6131 × X + 132.3	0.3292 × X + 16.71	−0.02538 × X + 9.592	−0.03765 × X + 11.90	−0.01227 × X + 2.306
	R^2^	0.0418	0.0262	0.1113	0.0766	0.0540	0.0113
	1 − β	0.2137	0.1497	0.4987	0.3598	0.2649	0.0914
							
Right YBT-LQ PM (cm)	R	−0.4504	−0.3487	0.5788	−0.3173	−0.2997	−0.1682
	95% CI for R	−0.6844 to −0.1324	−0.6144 to −0.01194	0.2991 to 0.7668	−0.5920 to 0.02332	−0.5791 to 0.04285	−0.4791 to 0.1802
	*p*	0.0075	0.0433	0.0003	0.0674	0.0851	0.3416
	Y	−0.4264 × X + 73.46	−1.1667 × X + 176.7	0.5038 × X + 2.249	−0.02567 × X + 9.696	−0.04282 × X + 12.42	−0.01716 × X + 2.721
	R^2^	0.2029	0.1216	0.3350	0.1007	0.0898	0.2830
	1 − β	0.0914	0.5375	0.9616	0.4574	0.4139	0.1584
							
Right YBT-LQ-PL (cm)	R	−0.4689	−0.4073	0.5583	−0.4017	−0.3822	−0.2170
	95% CI for R	−0.6966 to −0.1554	−0.6552 to −0.08023	0.2714 to 0.7541	−0.6514 to −0.07357	−0.6379 to −0.05058	−0.5172 to 0.1308
	*p*	0.0052	0.0168	0.0006	0.0185	0.0257	0.2177
	Y	−0.3939 × X + 71.91	−1.209 × X + 182.8	0.4312 × X + 6.784	−0.02884 × X + 9.829	0.04847 × X + 12.97	−0.01963 × X + 2.958
	R^2^	0.2199	0.1659	0.3117	0.1614	0.1461	0.0471
	1 − β	0.8202	0.6856	0.9453	0.6720	0.6223	0.2359
							
Left YBT-LQ-ANT (cm)	R	−0.1709	−0.2161	0.3689	−0.2786	−0.2373	−0:1115
	95% CI for R	−0.4813 to 0.1775	−0.5165 to 0.1317	0.03516 to 0.6287	−0.5637 to 0.06575	−0.5327 to 0.1097	−0.4333 to 0.2355
	*p*	0.3339	0.2197	0.0318	0.1106	0.1766	0.5300
	Y	−0.2034 × X + 55.84	−0.9082 × X + 154.6	0.4037 × X + 10.94	−0.02833 × X + 9.829	−0.04262 × X + 12.30	−0.01430 × X + 2.466
	R^2^	0.0292	0.0467	0.1361	0.0776	0.0563	0.0124
	1 − β	0.1621	0.2343	0.5894	0.3637	0.2746	0.0959
							
Left YBT-LQ-PM (cm)	R	−0.2893	−0.2479	0.4581	−0.3580	−0.3049	−0.1432
	95% CI for R	−0.5715 to 0.05421	−0.5407 to 0.09852	0.1419 to 0.6895	−0.6210 to −0.02261	−0.5829 to 0.03714	−0.4592 to 0.2049
	*p*	0.0971	0.1575	0.0064	0.0376	0.0796	0.4190
	Y	−0.2881 × X + 62.98	−0.8721 × X + 154.5	0.4195 × X + 8.520	−0.03047 × X + 10.08	−0.04583 × X + 12.67	−0.01537 × X + 2.591
	R^2^	0.0837	0.0615	0.2100	0.1282	0.0929	0.0205
	1 − β	0.3888	0.2962	0.2962	0.5614	0.4266	0.1273
							
Left YBT-LQ-PL (cm)	R	−0.3754	−0.2724	0.5015	−0.4039	−0.3407	−0.1571
	95% CI for R	−0.6332 to −0.04264	−0.5591 to 0.07246	0.1967 to 0.7179	−0.6529 to −0.07614	−0.6087 to −0.002886	−0.4703 to 0.1912
	*p*	0.0287	0.1191	0.0025	0.0178	0.0486	0.3749
	Y	−0.3339 × X + 67.62	−0.8557 × X + 156.1	0.4101 × X + 7.890	−0.03069 × X + 10.20	−0.04574 × X + 12.81	−0.01505 × X + 2.616
	R^2^	0.1409	0.0742	0.2515	0.1631	0.1161	0.0247
	1 − β	0.6061	0.3496	0.8764	0.6773	0.5169	0.1439
							
TUG_DTC (score)	R	0.4330	0.5927	−0.5299			
	95% CI for R	0.1111 to 0.6727	0.3184 to 0.7755	−0.7361 to −0.2335			
	*p*	0.0105	0.0002	0.0013			
	Y	4.020 × X + 35.09	19.44 × X + 59.90	−4.523 × X + 47.26			
	R^2^	0.1875	0.3513	0.2808			
	1 − β	0.7456	0.9705	0.9154			

Legend: HGS: HandGrip Strength test; 30sACT: 30-s Arm Curl Test; QMIS: Quadriceps Maximal Isometric Strength; 30sCST: 30-s Chair Stand Test; 6MWT: 6 Minutes Walking Test; YBT-LQ-CS: Y-Balance Test-Lower Quarter-Composite Score; YBT-LQ-ANT: Y-Balance Test-Lower Quarter-Anterior; YBT-LQ-PM: Y-Balance Test-Lower Quarter-PosteroMedial; YBT-LQ-PL: Y-Balance Test-Lower Quarter-PosteroLateral; TUG_ST: Timed Up & Go test Single Task; TUG_DT: Timed Up & Go test Dual Task; TUG_DTC: Timed Up & Go Test Dual Task Component; TMT-A: Trail Making Test A; TMT-B: Trail Making Test B; DSST: Digit Symbol Substitution Test. R: Pearson’s correlation coefficient; 95% CI for R: 95% confidence interval for R; *p*: *p*-value; Y: dependent variable in regression models; R^2^: coefficient of determination (effect size; proportion of shared variance); 1 − β: post hoc statistical power calculated using G*Power 3.1.

## Data Availability

The data presented in this study are available on reasonable request from the corresponding author. The data are not publicly available due to privacy and ethical restrictions related to participant confidentiality.

## Supplementary Materials

The following supporting information can be downloaded at: https://www.mdpi.com/article/10.3390/sports13120429/s1, Table S1: Pearson correlation coefficients (R), unadjusted *p*-valeus (*p*), and FDR-adjusted *p*-values (q-value) for associations between physical performance and cognitive outcomes. (N = 34).

## Abbreviations

6MWT	Six-Minute Walk Test
30sACT	30-Second Arm Curl Test
30sCST	30-Second Chair Stand Test
ANT	Anterior (direction in Y-Balance Test)
BMI	Body Mass Index
CI	Confidence Interval
CS	Composite Score
DSST	Digit Symbol Substitution Test
DT	Dual Task
DTC	Dual Task Cost
HGS	Handgrip Strength
LL	Limb Length
PL	Posterolateral (direction in Y-Balance Test)
PM	Posteromedial (direction in Y-Balance Test)
QMIS	Quadriceps Maximal Isometric Strength
SD	Standard Deviation
ST	Single Task
TMT-A	Trail Making Test Part A
TMT-B	Trail Making Test Part B
TUG	Timed Up and Go Test
YBT-LQ	Y-Balance Test—Lower Quarter
