# Supplementary material for: Cognition, Physical Performance, and Fall-Related Mobility Outcomes in Healthy Older Adults: A Cross-Sectional Study
Source: Sports (Basel). 2025 Dec 3;13(12):429. doi: 10.3390/sports13120429 (PMC12737262; doi:10.3390/sports13120429)
Supplement: Supplementary file 1 [file sports-13-00429-s001.zip › sports-3933055-supplementary.pdf]

**Supplementary Table S1. Pearson correlation coefficients (R), unadjusted p-valeus (P), and FDR-adjusted p-values (q-value) for associations between physical performance and cognitive outcomes. (N.=34).**

| | | TMT-A (sec) | TMT-B (sec) | DSST (score) | TUG_ST (sec) | TUG_DT (sec) | TUG_DTC (score) |
|---|---|---|---|---|---|---|---|
| Right HGS (kg) | R | −0.03368 | −0.1411 | 0.07658 | −0.3722 | −0.4563 | −0.3442 |
| | P | 0.8500 | 0.4262 | 0.6669 | 0.0302 | 0.0067 | 0.0462 |
| | q-value | 0.7438 | 0.5594 | 0.7002 | 0.0793 | 0.0352 | 0.0809 |
| Left HGS (kg) | R | −0.1971 | −0.1946 | 0.1647 | −0.3277 | −0.4201 | −0.3288 |
| | P | 0.2638 | 0.2702 | 0.3519 | 0.0585 | 0.0134 | 0.0576 |
| | q-value | 0.3405 | 0.3405 | 0.3695 | 0.1229 | 0.0844 | 0.1229 |
| 30sACT (n° reps) | R | −0.3655 | −0.3645 | 0.2782 | −0.3504 | −0.1689 | 0.04119 |
| | P | 0.0335 | 0.0341 | 0.1112 | 0.0422 | 0.3395 | 0.8171 |
| | q-value | 0.0886 | 0.0886 | 0.1751 | 0.0886 | 0.4278 | 0.8580 |
| Right QMIS (N) | R | −0.3141 | −0.3514 | 0.2489 | −0.2059 | −0.4237 | −0.4305 |
| | P | 0.0704 | 0.0416 | 0.1558 | 0.2427 | 0.0125 | 0.0110 |
| | q-value | 0.0739 | 0.0582 | 0.1309 | 0.1699 | 0.0263 | 0.0263 |
| Left QMIS (N) | R | −0.3222 | −0.3464 | 0.2191 | −0.02172 | −0.3177 | −0.2730 |
| | P | 0.0632 | 0.0448 | 0.2132 | 0.2173 | 0.0671 | 0.1182 |
| | q-value | 0.1409 | 0.1409 | 0.2282 | 0.2282 | 0.1409 | 0.1862 |
| 30sCST (n° reps) | R | −0.2985 | −0.2821 | 0.1613 | −0.3666 | −0.2811 | −0,1031 |
| | P | 0.0864 | 0.1059 | 0.3620 | 0.0330 | 0.1073 | 0.5616 |
| | q-value | 0.1690 | 0.1690 | 0.4561 | 0.1690 | 0.1690 | 0.5897 |
| 6MWT (m) | R | −0.3214 | −0.3450 | 0.3460 | −0.5138 | −0.3742 | −0.1169 |
| | P | 0.0638 | 0.0457 | 0.0451 | 0.0019 | 0.0292 | 0.5104 |
| | q-value | 0.0670 | 0.0600 | 0.0600 | 0.0100 | 0.0600 | 0.4466 |
| Right YBT-LQ-CS (score) | R | −0.3333 | −0.2738 | 0.4895 | −0.2371 | −0.2221 | −0.1232 |
| | P | 0.0541 | 0.1171 | 0.0033 | 0.1770 | 0.2068 | 0.4875 |
| | q-value | 0.1420 | 0.2049 | 0.0173 | 0.2171 | 0.2171 | 0.4266 |
| Left YBT-LQ-CS (score) | R | −0.1561 | −0.1679 | 0.3708 | −0.2573 | −0.1947 | −0.06868 |
| | P | 0.3760 | 0.3426 | 0.0308 | 0.1417 | 0.2699 | 0.6995 |
| | q-value | 0.7463 | 0.4763 | 0.1940 | 0.4464 | 0.4763 | 0.7345 |
| Right YBT-LQ-ANT (cm) | R | −0.2044 | −0.1617 | 0.3336 | −0.2769 | −0.2324 | −0.1061 |
| | P | 0.2462 | 0.3608 | 0.0538 | 0.1129 | 0.1859 | 0.5502 |
| | q-value | 0.3878 | 0.4546 | 0.3890 | 0.3556 | 0.3878 | 0.5777 |
| Right YBT-LQ PM (cm) | R | −0.4504 | −0.3487 | 0.5788 | −0.3173 | −0.2997 | −0.1682 |
| | P | 0.0075 | 0.0433 | 0.0003 | 0.0674 | 0.0851 | 0.3416 |
| | q-value | 0.0158 | 0.0606 | 0.0013 | 0.0708 | 0.0715 | 0.2391 |
| Right YBT-LQ-PL (cm) | R | −0.4689 | −0.4073 | 0.5583 | −0.4017 | −0.3822 | −0.2170 |
| | P | 0.0052 | 0.0168 | 0.0006 | 0.0185 | 0.0257 | 0.2177 |
| | q-value | 0.0027 | 0.0049 | 0.0006 | 0.0049 | 0.0054 | 0.0381 |
| Left YBT-LQ-ANT (cm) | R | −0.1709 | −0.2161 | 0.3689 | −0.2786 | −0.2373 | −0.1115 |
| | P | 0.3339 | 0.2197 | 0.0318 | 0.1106 | 0.1766 | 0.5300 |
| | q-value | 0.4207 | 0.3460 | 0.2003 | 0.3460 | 0.3460 | 0.5565 |
| Left YBT-LQ PM (cm) | R | −0.2893 | −0.2479 | 0.4581 | −0.3580 | −0.3049 | −0.1432 |
| | P | 0.0971 | 0.1575 | 0.0064 | 0.0376 | 0.0796 | 0.4190 |
| | q-value | 0.1274 | 0.1654 | 0.0336 | 0.0987 | 0.1274 | 0.3666 |
| Left YBT-LQ-PL (cm) | R | −0.3754 | −0.2724 | 0.5015 | −0.4039 | −0.3407 | −0.1571 |
| | P | 0.0287 | 0.1191 | 0.0025 | 0.0178 | 0.0486 | 0.3749 |
| | q-value | 0.0502 | 0.1251 | 0.0131 | 0.0467 | 0.0638 | 0.3280 |
| TUG_DTC (score) | R | 0.4330 | 0.5927 | −0.5299 | | | |
| | P | 0.0105 | 0.0002 | 0.0013 | | | |
| | q-value | 0.0110 | 0.0006 | 0.0020 | | | |

Legend: HGS: HandGrip Strength test; 30sACT: 30-second Arm Curl Test; QMIS: Quadriceps Maximal Isometric Strength; 30sCST: 30-second Chair Stand Test; 6MWT: 6 Minutes Walking Test; YBT-LQ-CS: Y-Balance Test-Lower Quarter-Composite Score; YBT-QL-ANT: Y-Balance Test-Lower Quarter-Anterior; YBT-LQ-PM: Y-Balance Test-Lower Quarter-PosteroMedial; YBT-LQ-PL: Y-Balance Test-Lower Quarter-PosteroLateral; TUG_ST: Timed Up & Go test Single Task; TUG_DT: Timed Up & Go test Dual Task; TUG_DTC: Timed Up & Go Test Dual Task Component; TMT-A: Trail Making Test A; TMT-B: Trail Making Test B; DSST: Digit Symbol Substitution Test.
